# Oocyte and zygote development potential in minimal stimulation, natural cycle and conventionally stimulated IVF: an international multi-centre retrospective cohort study

**DOI:** 10.1007/s10815-025-03508-3

**Published:** 2025-05-28

**Authors:** Isotta Martha Magaton, Ikbale Siercks, Martina Nordin, Roxana Maria Popovici, Eva Maria Boogen, Stefan Eisenhardt, Natalie Reeka, Jan-Simon Lanowski, Marie Roumet, Michael von Wolff

**Affiliations:** 1 Division of Gynaecological Endocrinology and Reproductive Medicine, Inselspital University Hospital, Friedbuehlstrasse 19, 3010 Bern, Switzerland; 2 Institute for Gynaecological Endocrinology and Reproductive Medicine, c/o YUNA Praxis AG, Brauerstrasse 95, 9016 St. Gallen, Switzerland; 3 Fertility Centre Baden AG, Mellingerstrasse 207, 5405 Baden, Switzerland; 4 Fertility Centre Kïz, Bayerstrasse 3, 80335 Munich, Germany; 5 Fertility Centre Bonner Bogen, Joseph-Schumpeter-Allee 1, 53227 Bonn, Germany; 6 Fertility Centre Frauenärzte, Heilbronnerstrasse 1, 74172 Neckarsulm/Heilbronn, Germany; 7 Fertility Centre Villa Kinderwunsch, Wörthstrasse 13, 89077 Ulm, Germany; 8 Fertility Centre and Human Genetics, Gartenstrasse 18-20, 31141 Hildesheim, Germany; 9 Department of Clinical Research, University of Bern, Mittelstrasse 43, 3012 Bern, Switzerland

**Keywords:** Natural cycle IVF, Minimal stimulation IVF, Fertilization rate, Oocyte development potential, Embryo development potential, Live birth rate, IVF-Naturelle

## Abstract

**Purpose:**

The aim of this research is to assess the development potential of oocytes and zygotes obtained from Natural cycle IVF (NC-IVF), different minimal stimulation IVF (Min stim-IVF) and conventionally stimulated IVF (cIVF) treatment protocols.

**Methods:**

International multi-centre retrospective cohort study including 1483 NC-IVF, 1208 Min stim-IVF, and 1892 cIVF cycles performed in 8 IVF centres between 01.2022 and 03.2023. The five Min stim-IVF protocols analysed included low dose clomiphene citrate, aromatase inhibitors, low dose (≤ 100 IU) gonadotropins, each alone or in combination. For each IVF protocol, we assessed and modelled the transition probabilities of (i) each observed oocyte developing into a zygote, (ii) each observed zygote developing into a gestational sac and (iii) each observed zygote developing into a live birth.

**Results:**

All modelled transition probabilities were found to be maximal in NC-IVF, minimal in cIVF with Min stim-IVF in between. The probability of transition from oocyte to zygote was 0.72 for NC-IVF, 0.56 to 0.65 for Min stim-IVF protocols and 0.54 for cIVF. The probability of transition from zygote to gestational sac was 0.21 for NC-IVF, 0.14 to 0.19 for Min stim-IVF and 0.09 for cIVF protocols and from zygote to live birth 0.16 for NC-IVF, 0.09 to 0.16 for Min stim-IVF and 0.06 for cIVF protocols.

**Conclusions:**

The transition probabilities of oocytes and zygotes appears to be higher in NC-IVF, followed by Min stim-IVF and then cIVF, suggesting that increasing dosages of gonadotropins might have a negative effect on oocyte/zygote development potential.

**Trial registration:**

Clinicaltrial.gov: NCT05125497. Registration date 03.11.2021.

**Supplementary Information:**

The online version contains supplementary material available at 10.1007/s10815-025-03508-3.

## Introduction

Ovarian stimulation with gonadotropins has become a key component of assisted reproductive technologies (ART). The aim of ovarian stimulation with gonadotropins is to maximize the number of oocytes retrieved, allowing embryo selection and the surplus embryos to be cryopreserved. Although conventional IVF (cIVF) protocols are associated with good clinical outcomes, they are also associated with some patient discomfort, stress, intensive monitoring, anaesthesia, high cost and risk of complications.

Because of these disadvantages and due to the desire of many women to undergo Natural cycle IVF (NC-IVF) or minimal stimulation IVF (Min stim-IVF), IVF treatments without any or with minimal stimulation have experienced a revival in recent years. NC-IVF was proposed as a patient-friendly and also quite effective treatment option in patients with good prognosis for IVF treatment success [1–3]. Indeed, NC-IVF has been shown to generate oocytes with development potential better than cIVF treatments [2, 4, 5]. The proportion of mature oocytes, the fertilisation rate, the morphology of the cleavage stage embryos [4] and the live birth rates per transfer were found to be higher in NC-IVF compared to cIVF treatments [2].

However, even though the concept of natural follicular growth is very attractive, its disadvantages are also obvious [6, 7]. As only one follicle can be aspirated, the transfer rate per cycle is reduced [4]. Therefore, in cases with less favorable prognostic criteria, such as advanced maternal age (> 35 years old), success rates drop sharply [8–10].

As a result, several Min stim-IVF protocols have been developed to combine the advantages of NC-IVF with the advantages of stimulated cycles to achieve a higher oocyte yield. Min stim-IVF was developed as a compromise of both treatment options with the aim to benefit from advantages of either treatment modality with the aim to achieve cost-effective, less stressful and more patient-friendly regimens [6, 11–13]. Min stim-IVF protocols are usually based on oral compounds such as clomiphene citrate or aromatase inhibitors alone or in combination with low-dose gonadotropins (≤ 100 IU/day). Oral compounds also reduce the risk of premature ovulation [14], thus reducing the need for additional Gonadotropin releasing hormone (GnRH) antagonist injections.

As the fertilization rate and embryo morphology of oocytes generated by NC-IVF in fresh cycles has been shown to be better than that of cIVF [4], the question arises whether minimal stimulation also has some effect on the oocyte development potential. Such an effect might have implications for patient counselling and treatment strategies.

We therefore use data from a large-scale multi-centre cohort to assess the development potential of oocytes and zygotes in fresh cycles obtained from NC-IVF and cIVF, and several Min stim-IVF protocols.

## Materials and methods

### Study design

We conducted an international multi-centre retrospective cohort study of women undergoing different IVF therapies between January 2022 and March 2023. IVF therapies included NC-IVF, Min Stim-IVF and cIVF. Five fertility centres in Germany and three fertility centres in Switzerland participated in the study. The eight participating centres were specialized in NC-IVF and Min stim-IVF and are part of the “IVF-Naturelle” network (www.IVF-Naturelle.com), all performing the same treatment protocols. The Division of Gynaecological Endocrinology and Reproductive Medicine, University Women's Hospital, Inselspital, Bern, acted as a leading centre and planned and organized the study. Detailed information regarding setting, patient characteristics and main inclusion and exclusion criteria were previously published [13].

Briefly, eligible women underwent the below mentioned IVF therapies and planned to have a fresh transfer. Women with the cause of infertility “single” or “same-sex couple” were excluded from the analysis. We also excluded from the analysis cycles where the fertilization method was mixed or unknown.

After the diagnostic phase and IVF-treatment counselling, an IVF protocol was assigned to each patient according to specific characteristics such as cause of infertility, female age, anti-Müllerian hormone (AMH) concentration, body mass index, accessibility of the ovaries, and sensitivity to pain. Personal preferences were also considered. A change of IVF stimulation protocols was always possible during the process of IVF treatment. The study was registered at Clinicaltrial.gov, study number NCT05125497, and the “Strengthening the Reporting of Observational Studies in Epidemiology” (STROBE) criteria were followed.

#### Objectives

The main objective of the study was to assess the development potential of oocytes and zygotes derived from NC-IVF, Min stim-IVF and cIVF protocols by estimating the probabilities of (i) each observed oocyte developing into a zygote, (ii) each observed zygote developing into a gestational sac and (iii) each observed zygote developing into a live birth.

#### Protocols

Detailed information regarding NC-IVF and Min stim-IVF protocols was described in another publication [13]. The three IVF therapies are briefly described here.

1. Natural cycle IVF (NC-IVF)

NC-IVF was defined as IVF treatment without any ovarian stimulation to induce follicular growth. A single GnRH antagonist injection was given if necessary, and ovulation was induced by human chorionic gonadotropin (hCG, 5000 IU).

2. Minimal stimulation IVF (Min stim-IVF)

Min stim-IVF was defined as IVF treatment with ≤ 100 IU human menopausal gonadotropin (hMG), recombinant human follicle-stimulating hormone (FSH) per day, clomiphene citrate (CC, ≤ 25 mg/d) and aromatase inhibitors (AI, ≤ 5 mg/d), given each alone or in combination. Five different standardised Min stim-IVF protocols were developed and used by all participating centres, and hCG (5000 IU) was used to induce ovulation in all protocols and centres.

##### Min stim-IVF with CC

25 mg of CC per day was started around day 4 of the cycle until the day of the ovulation trigger (Protocol “CC-IVF”).

##### Min stim-IVF with AI

5 mg of letrozole per day was started around day 4 of the cycle for 5 days (Protocol “AI-IVF”).

##### Min stim-IVF with hMG or FSH

75–100 IU of hMG or FSH per day was started around day 4 of the cycle until the day before the ovulation trigger was given (Protocol “hMG/FSH-IVF”).

##### Min stim-IVF with CC and hMG or FSH

The “CC-IVF” protocol was supplemented by 75–100 IU/day of hMG or FSH from around day 4 of the cycle until the day before ovulation was induced (Protocol “CC + hMG/FSH-IVF”).

##### Min stim-IVF with AI and hMG or FSH

The “AI-IVF” protocol was supplemented by 75–100 IU/day of hMG or FSH from around day 4 of the cycle until the day before ovulation was induced (Protocol “AI + hMG/FSH-IVF”).

3. Conventional IVF (cIVF)

cIVF was defined as any IVF treatment with hMG or FSH stimulation with dosages of 150 to 300 IU per day. LH surge was controlled by GnRH agonists or antagonists. Ovulation was induced by 5000 IU of hCG in all cycles.

For all the three types of IVF, the ovarian response was monitored by transvaginal ultrasound and assessment of serum luteinising hormone (LH) and oestradiol (E2) concentrations analysed by electrochemiluminescence. Oocyte pick-up was performed 36 h after hCG injection. The fertilization technique used was either standard IVF or intracytoplasmic sperm injection (ICSI). Embryo transfer was performed on day 2, 3 or 5, depending on the protocol of the participating centre, the cycle outcome and the preference of the patient. Surplus embryos were cryopreserved.

### Statistical analysis

#### Missing data due to cryopreservation

Oocyte development was coded as missing in case of oocyte cryopreservation, and zygote development was coded as missing in case of zygote or embryo cryopreservation. Zygote development in cycles where cryopreserved embryos were used could not be assessed and was coded as missing as well.

### Population and cycle characteristics

Patient characteristics, cycle characteristics and clinical outcomes are described in tables stratified by IVF therapy protocols (NC-IVF, CC-IVF, AI-IVF, hMG/FSH-IVF, CC + hMG/FSH-IVF, AI + hMG/FSH-IVF, cIVF) without any formal statistical test. Categorical variables are summarized as number and proportion, continuous variables as median with interquartile range (IQR) and mean with standard deviation (SD).

### Transition probabilities

The observed proportions of oocytes developing into a zygote, of zygotes developing into a gestational sac and zygotes developing into a live birth are described in a table stratified by IVF therapy protocols without any formal statistical test. Results are presented as numbers and percentages with the associated 95% Wilson confidence intervals (CIs).

In addition to this first (crude) estimation, we estimated the transition probabilities from oocyte to zygote (oo-zyg), zygote to gestational sac (zyg-sac) and zygote to live birth (zyg-birth) using logistic regression mixed models. Each transition (i.e. oocytes into zygote, zygote into gestational sac and zygote into live birth) was modelled separately using a similar model. The model included the transition outcome (progression to the next stage, binary: yes or no) as dependent variable and the protocol (categorical) as independent variable and was adjusted for the effects of the duration of infertility (continuous, years), cause of infertility (categorical: female & male factor, female factor, male factor, idiopathic), fertilisation technique (IVF, ICSI), age (continuous, years), BMI (Body mass Index; continuous), and AMH level (categorical: < 1 ng/ml; ≥ 1 to < 2 ng/ml; ≥ 2 ng/ml). To account for the structure of the dataset and to take care of for arbitrary correlation between observations within patient and cycle, the model additionally included a random intercept for the patient and the cycles (nested within patient). Model results were used to derive the transition probabilities and associated 95% CIs for each protocol using marginal means. The results for different protocols were compared through post hoc pairwise comparisons, with *p*-values adjusted for multiple testing using the false discovery rate correction.

To assess the likelihood of a bias caused by embryo selection, the zygote development in cIVF cycles with and without cryopreservation (i.e. with and without the possibility for selection) was compared. Transition probabilities from zygote to gestational sac and live birth were modelled using mixed logistic regression. Models were similar to the one used for the main analysis with the additional inclusion of the effects of zygote and embryo cryopreservation. Transition probabilities of success in cIVF cycles with and without cryopreservation were derived using marginal means.

Results of the statistical analysis are represented in tables and graphically. All the statistical analyses were performed using R version 4.2.3.

### Ethical approval

Ethical approval for the three Swiss centres was given by the leading cantonal ethics committee of Bern for Switzerland (KEK ID 2021–01689, 04.10.2021) and for the five German centres by 4 German ethics committees in Baden-Württemberg (ID F-2024–068, 22.08.2024), Niedersachsen (ID Grae/080/2024, 30.08.2024), Bavaria (ID mb24043, 12.09.2024) and Nordrhein-Westfalen (ID 2024 194, 30.10.2024). Participants provided written informed consent.

##  Results

Baseline characteristics of patients stratified by IVF protocols are shown in Table 1. In total, we included 2380 women who underwent one or more than one IVF treatment protocol. As some patients underwent many IVF protocols, the overall population is not the sum of each IVF protocol population.

**Table 1:** Baseline characteristics of patients overall and stratified by IVF protocol

	**Overall**	**NC-IVF**	**CC-IVF**	**AI-IVF**	**hMG/FSH-IVF**	**CC + hMG/FSH-IVF**	**AI + hMG/FSH-IVF**	**cIVF**
**Characteristics**								
**Number of patients, *n***	2380^1^	578^2^	249^2^	103^2^	102^2^	157^2^	213^2^	1422^2^
**Number of cycles per patient, *s***								
Median [IQR]	1.0 [1.0, 2.0]	2.0 [1.0, 3.0]	1.0 [1.0, 2.0]	1.0 [1.0, 2.0]	1.0 [1.0, 2.0]	1.0 [1.0, 1.0]	1.0 [1.0, 1.0]	1.0 [1.0, 2.0]
Range, [min, max]	[1.0, 12.0]	[1.0, 10.0]	[1.0, 10.0]	[1.0, 4.0]	[1.0, 11.0]	[1.0, 4.0]	[1.0, 6.0]	[1.0, 8.0]
**Female age at 1**^st^** OPU, years**								
Mean (± SD)	36.0 (± 4.4)	36.6 (± 3.8)	36.1 (± 4.5)	35.9 (± 4.7)	37.2 (± 3.7)	37.1 (± 4.4)	36.9 (± 4.5)	35.6 (± 4.4)
Range, [min, max]	[21.0, 48.0]	[23.0, 46.0]	[24.0, 46.0]	[24.0, 46.0]	[27.0, 45.0]	[22.0, 46.0]	[22.0, 46.0]	[21.0, 48.0]
**BMI (Kg/m**^**2**^**)**								
Mean (± SD)	24.1 (± 4.7)	23.0 (± 4.1)	23.9 (± 4.7)	23.4 (± 4.4)	23.0 (± 4.7)	24.2 (± 4.9)	24.2 (± 4.8)	24.5 (± 4.9)
Range, [min, max]	[15.4, 46.7]	[15.8, 44.2]	[15.4, 40.6]	[16.2, 39.4]	[15.4, 44.2]	[16.8, 44.2]	[16.3, 42.7]	[15.4, 46.7]
Missings	9	2	0	1	0	0	0	7
**Duration of infertility, years**								
Median [IQR]	3.0 [2.0, 4.0]	3.0 [2.0, 4.0]	3.0 [2.0, 4.0]	3.0 [2.0, 4.0]	3.0 [2.0, 4.0]	3.0 [2.0, 5.0]	3.0 [2.0, 4.0]	3.0 [2.0, 4.0]
Range, [min, max]	[1.0, 17.0]	[1.0, 15.0]	[1.0, 10.0]	[1.0, 10.0]	[1.0, 10.0]	[1.0, 15.0]	[1.0, 13.0]	[1.0, 17.0]
Missings	2	0	0	0	0	0	0	2
**Causes of infertility, *n* (%)**								
Male factor	948 (40%)	156 (27%)	95 (38%)	26 (25%)	36 (35%)	46 (29%)	59 (28%)	673 (47%)
Female factor	595 (25%)	174 (30%)	43 (17%)	28 (27%)	27 (26%)	46 (29%)	73 (34%)	314 (22%)
Male and female	490 (21%)	111 (19%)	71 (29%)	29 (28%)	18 (18%)	37 (24%)	52 (24%)	269 (19%)
Idiopathic	347 (15%)	137 (24%)	40 (16%)	20 (19%)	21 (21%)	28 (18%)	29 (14%)	166 (12%)
**AMH level (ng/ml)**								
Median [IQR]	2.0 [0.9, 3.6]	1.8 [0.7, 3.1]	2.0 [1.0, 3.9]	1.8 [0.4, 3.5]	1.6 [0.8, 3.3]	1.4 [0.6, 3.2]	1.5 [0.4, 3.1]	2.1 [1.1, 3.9]
Range, [min, max]	[0.0, 21.0]	[0.0, 15.0]	[0.0, 16.8]	[0.0, 12.5]	[0.0, 21.0]	[0.0, 14.1]	[0.0, 15.0]	[0.0, 21.0]
< 1	610 (26%)	190 (33%)	61 (25%)	40 (39%)	31 (30%)	55 (35%)	84 (39%)	292 (21%)
≥ 1 to < 2	540 (23%)	131 (23%)	51 (21%)	14 (14%)	27 (26%)	41 (26%)	40 (19%)	343 (24%)
≥ 2	1212 (51%)	251 (44%)	135 (55%)	48 (47%)	44 (43%)	60 (38%)	89 (42%)	778 (55%)
Missings	18	6	2	1	0	1	0	9
**Any embryo transfer without clinical pregnancy since the last birth, *n* (%)**								
Yes	499 (21%)	116 (20%)	27 (11%)	26 (25%)	28 (27%)	40 (25%)	63 (30%)	295 (21%)
No	1881 (79%)	462 (80%)	222 (89%)	77 (75%)	74 (73%)	117 (75%)	150 (70%)	1127 (79%)

^1^Number of patients

^2^Number of patients per protocol. As some patients underwent many IVF protocols, the overall population is not the sum of each IVF protocol population

*AI*, aromatase inhibitor; AMH, anti-Müllerian hormone; *BMI*, body mass index; *CC*, clomiphene citrate; *cIVF*, conventional IVF; *FSH*, follicle-stimulating hormone; *hMG*, human menopausal gonadotropin; *IQR*, interquartile range; *IVF*, in vitro* fertilization*; *min*, minimum; *max*, maximum; *OPU*, oocyte pick-up; *SD*, standard deviation

The analysis included a total of 4583 IVF cycles. Of these, 1483 were NC-IVF cycles, 1208 Min stim-IVF cycles and 1892 cIVF cycles. Min stim-IVF cycles were represented as follows: 434 cycles of CC-IVF, 149 cycles of AI-IVF, 153 cycles of hMG/FSH-IVF, 200 cycles of CC + hMG/FSH-IVF and 272 cycles of AI + hMG/FSH-IVF.

The observed reproductive outcomes for each of these cycles are shown in Table 2. Since oocytes and/or zygotes and embryos were cryopreserved, the results shown in Table 2 may represent an underestimation of the real potential of IVF protocols. Table 3 shows the descriptive observed proportions of oocytes that developed into a zygote, zygotes that developed into a gestational sac and zygotes that developed into a live birth. Additionally, the table shows the amount of missing data, primarily due to cryopreservation of the material. Detailed cycle characteristics are shown in Supplementary Table S1. The median number of oocytes retrieved per cycle by all 2444 participants was 2.0, and the mean ± standard deviation (SD) was 4.5 ± 5.5.

**Table 2:** Overall clinical outcomes and outcomes per protocol and per at least 1 fresh embryo transfer

	**Overall**	**NC-IVF**	**CC-IVF**	**AI-IVF**	**hMG/FSH-IVF**	**CC + hMG/FSH-IVF**	**AI + hMG/FSH-IVF**	**cIVF**
**Cycles,* n***	4583	1483	434	149	153	200	272	1892
**Premature ovulations, *n* (%)**	171 (3.6%)	125 (8.4%)	15 (3.5%)	3 (2.0%)	10 (6.5%)	4 (2.0%)	5 (1.8%)	9 (0.5%)
**Cycles with at least 1 fresh embryo transfer, *n* (%)**	3057 (67%)	791 (53%)	275 (63%)	75 (50%)	102 (67%)	152 (76%)	214 (79%)	1448 (77%)
**Unknown, *n***	4	3	0	0	0	0	0	1
**Number of transferred embryos**								
Median [IQR]	1.0 [0.0, 2.0]	1.0 [1.0, 1.0]	1.0 [1.0, 1.0]	1.0 [1.0, 1.0]	1.0 [1.0, 1.0]	1.0 [1.0, 2.0]	1.0 [1.0, 2.0]	1.0 [1.0, 2.0]
Mean (± SD)	1.3 (± 0.5)	1.0 (± 0.2)	1.1 (± 0.3)	1.2 (± 0.4)	1.3 (± 0.5)	1.4 (± 0.5)	1.5 (± 0.5)	1.4 (± 0.5)
**Day of embryo transfer**								
Median [IQR]	3.0 [3.0, 5.0]	3.0 [3.0, 4.0]	3.0 [2.0, 4.0]	2.0 [2.0, 3.0]	3.0 [2.0, 5.0]	3.0 [2.0, 5.0]	3.0 [3.0, 5.0]	5.0 [3.0, 5.0]
Mean (± SD)	3.6 (± 1.2)	3.4 (± 1.1)	3.1 (± 1.1)	2.6 (± 1.0)	3.5 (± 1.2)	3.4 (± 1.2)	3.6 (± 1.3)	3.9 (± 1.3)
**Cycles with at least 1 fresh embryo transfer, *n***	3057	791	275	75	102	152	214	1448
At least 1 gestational sac, *n* (%)	926 (30%)	201 (25%)	57 (21%)	19 (25%)	24 (24%)	44 (29%)	68 (32%)	513 (35%)
Unknown	3	2	0	0	0	1	0	0
At least 1 live birth, *n* (%)	707 (23%)	149 (19%)	47 (17%)	16 (21%)	19 (19%)	27 (18%)	50 (23%)	399 (28%)
Unknown	3	2	0	0	0	1	0	0
Cycles with at least 1 gestational sac, *n*	926	201	57	19	24	44	68	513
Miscarriages, *n* (%)	235 (25%)	50 (25%)	10 (18%)	4 (21%)	6 (25%)	22 (50%)	21 (31%)	122 (24%)

*AI*, aromatase inhibitor; *CC*, clomiphene citrate; *cIVF*, conventional IVF; *FSH*, follicle-stimulating hormone; *hMG*, human menopausal gonadotropin; *IQR*, interquartile range; *IVF*, in vitro* fertilization*; *NC-IVF*, Natural cycle IVF; *SD*, standard deviation

**Table 3:** Transition probability of IVF outcomes overall and stratified by IVF protocol

	**Overall** **[95% CI]**^**1,2**^	**NC-IVF** **[95% CI]**^**1,2**^	**CC-IVF** **[95% CI]**^**1,2**^	**AI-IVF** **[95% CI]**^**1,2**^	**hMG/FSH-IVF** **[95% CI]**^**1,2**^	**CC + hMG/FSH-IVF** **[95% CI]**^**1,2**^	**AI + ** **hMG/FSH-IVF** **[95% CI]**^**1,2**^	**cIVF** **[95% CI]**^**1,2**^
Characteristics								
Number of oocytes, *n*	19,812	1291	635	194	392	593	977	15,730
Number of cryopreserved oocytes, *n*	68	5	0	0	0	0	2	61
Number of zygotes, n	11,162	935	404	109	250	369	626	8469
Number of cryopreserved zygotes, *n*	2980	15	32	4	41	49	103	2736
Oocytes’ development into zygotes	57% [56%,57%]	73% [70%,75%]	64% [60%,67%]	56% [49%,63%]	64% [59%,68%]	62% [58%,66%]	64% [61%,67%]	54% [53%,55%]
Zygotes’ development into gestational sacs	12% [12%,13%]	22% [20%,25%]	16% [12%,20%]	20% [13%,29%]	13% [9.0%,18%]	16% [12%,20%]	12% [12%,18%]	10% [9.4%,11%]
Zygotes’ development into live births	9.3% [8.7%,10%]	16% [14%, 19%]	13% [9.9%, 17%]	16% [10%, 24%]	9.6% [6.3%,14%]	8.8% [6.1%,12%]	11% [8.3%,14%]	7.8% [7.1%,8.5]

^1^*n*, %

^2^*CI* Wilson confidence interval

*AI*, aromatase inhibitor; *CC*, clomiphene citrate; *CI*, Wilson confidence interval; *cIVF*, conventional IVF; *FSH*, follicle-stimulating hormone; *hMG*, human menopausal gonadotropin; *IVF*, in vitro* fertilization*; *NC-IVF*, natural cycle IVF

**Fig. 1:**
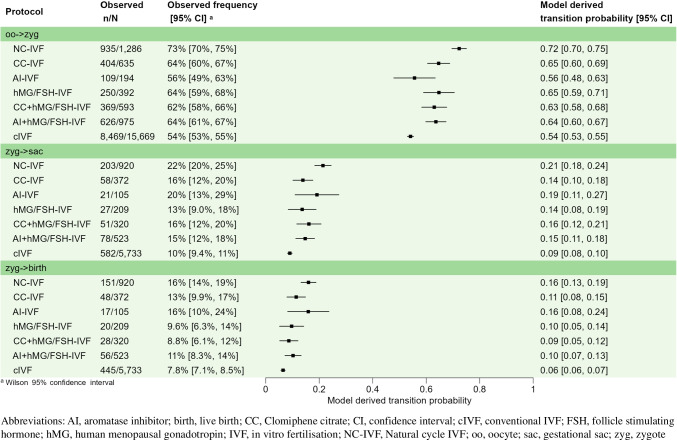
Forest plot: observed and predicted transition probabilities from oocyte to zygote, from zygote to gestational sac and live birth and per IVF protocol

The transition probabilities derived from the logistics regression models, presented in Supplementary Table S2, are very close to the crude observations presented in Table 3 and are shown in Fig. 1. The probability of transition from oocyte to zygote was 0.72 (95% CI, 0.70–0.75) for NC-IVF, 0.56 to 0.65 for Min stim-IVF and 0.54 (95% CI, 0.53–0.55) for cIVF protocols (Fig. 1). The difference in the transition probabilities estimated in the NC-IVF and cIVF protocols was found to be significant (risk difference − 0.18 (95% CI, − 0.21, − 0.15), *p* ≤ 0.001, Supplementary Figure S2).

The probability of transition from zygote to gestational sac was 0.21 for NC-IVF (95% CI, 0.18–0.24), 0.14 to 0.19 for Min stim-IVF and 0.09 (95% CI, 0.08–0.10) for cIVF protocols (Fig. 1). The difference in the transition probabilities estimated in the NC-IVF and cIVF protocols was found to be significant (risk difference − 0.12 (95% CI, − 0.16, − 0.09), *p* < 0.001, Supplementary Figure S3).

The probability of transition from zygote to live birth was 0.16 (95% CI 0.13–0.19) for NC-IVF, 0.09 to 0.16 for Min stim-IVF and 0.06 (95% CI, 0.06–0.07) for cIVF protocols (Fig. 1). Again, the difference in the transition probabilities estimated in the NC-IVF and cIVF protocols was found to be significant (risk difference − 0.10 (95% CI, − 0.12, − 0.07), *p* < 0.001, Supplementary Figure S4).When analysing the transition probability of cIVF’s zygotes, we observe a significant effect of the cryopreservation of embryos (Supplementary Table S3). Namely, the development of a zygote into a gestational sac and into a live birth are both more likely to occur in cycles with embryo cryopreservation than in cycles without (OR = 1.61 (95% CI, 1.29, 2.01), *p* < 0.001 and OR = 1.51 (95% CI 1.17, 1.96), *p* = 0.002, respectively). Model-based estimations of the probabilities of transition of a zygote into a gestational sac are 0.13 (95% CI 0.11–0.15) and 0.08 (95% CI 0.07–0.09), in cycles with and without embryo cryopreservation, respectively. For the transition of a zygote into a live birth, estimations are 0.09 (95% CI 0.07–0.11) and 0.06 (95% CI 0.05–0.07), in cycles with and without embryo cryopreservation, respectively.

## Discussion

This study describes the development potential of oocytes and zygotes in fresh cycles obtained by Min Stim-IVF, NC-IVF and cIVF. Oocytes obtained by NC-IVF have the highest potential to develop into a zygote, those obtained by cIVF have the lowest and those obtained by Min stim-IVF are in between. The same relationships were found for the transitions of zygotes into gestational sacs and live births. To the best of our knowledge, this is the first study to assess the development potential of oocytes and zygotes in Min stim-IVF treatments. Additionally, the study extends our previous findings that the fertilization potential and embryo development potential in fresh cycles is higher in NC-IVF compared to cIVF treatments [2, 4, 15].

The strength of our study is the large number of IVF cycles included, its multi-centre design which provides a kind of real-world data and the inclusion of NC-IVF, Min stim-IVF and cIVF treatment cycles. In addition, we report the development potential of IVF therapies not only based on oocytes but also on zygotes which includes more factors such as the fertilization potential of the oocyte.

The weakness of our study is that the data we have is only a description of the development potential in fresh cycles. Another substantial weakness is the bias due to embryo selection in stimulated cycles. As all participating centres transferred the best fresh embryos first and cryopreserved less favourable embryos, cycles with embryo selection were likely to have better and therefore overestimated “zyg-sac” and “zyg-birth” transition probabilities. This intuition was confirmed by our results, which showed a significantly higher transition probabilities of zygotes into gestational sacs and live births in cIVF cycles with embryo cryopreservation than in cycles without embryo cryopreservation. However, this bias cannot change the main conclusion of the study: transition probability are lower in stimulated cycles. This bias might even have led to an underestimation of the negative impact of stimulation on the transition probability.

Other weaknesses of our study include the fact that allocation of patients to the different IVF treatments was not randomized, also imposing some degree of bias. Moreover Min stim-IVF is a very heterogeneous, and non-standardized group of different medication’s protocols. Finally, some information regarding parameters that are known to influence oocyte and zygote development were not collected and could not be considered in our analysis. This is notably the case for the total dose of stimulation and the level of progesterone prior to the embryo transfer. Another confounding factor that we did not take into account was the trigger medication, which could also have had some effect on the oocytes by increasing the risk of spindle misalignment and chromosome missegregation [16]. Our analysis also does not take into account the effect of ovarian stimulation on endometrial function. According to some literature high oestrogen can affect endometrial receptivity [17]. In this regard, it’s also known that CC has some negative effects on the endometrium by suppressing endometrial oestrogen and progesterone receptor concentration. However, all participating centres used CC in a very low dosage of only 25 mg/d which has no effect on implantation rate [18].

According to our logistic regression analysis, AMH levels and women’s age have a significant effect on transition probabilities of zygote to gestational sacs and live birth, but not on the transition probability of oocytes to zygotes. A bias due to different fertilization techniques can be excluded as the transition probability was calculated per aspirated oocyte and not per metaphase II oocyte and as the calculation was adjusted for the fertilization technique. However, the maturity of oocytes is important and could be a marker of oocyte quality [19, 20].

Our results raise the question of whether high doses of exogenous gonadotropin might have a negative effect on oocyte quality, defined as the potential and ability to undergo meiotic maturation, fertilisation, embryonic development and clinical pregnancy [21]. However, studies on the effects of gonadotropin stimulation on oocyte and zygote development potential are limited [4, 22], and the reasons for the negative effects of gonadotropin stimulation could be diverse. We have previously shown that the oocyte maturation rates (89% vs. 82%, adjusted odds ratio (aOR) 1.79, *p* = 0.001) and fertilization rates (79% vs. 71%, aOR 1.62, *p* = 0.001) are higher with NC-IVF compared to cIVF [4].

Several other studies have analysed the association of the ovarian response and the oocyte development potential and/or the oocyte utilization rate [5, 23–29].

One of the first papers describing this association was published in 1997 [24]. The prospective study included cIVF cycles stratified into five groups according to the number of retrieved oocytes (group 1, 1 to 5 oocytes; group 5, > 20 oocytes). Although the pregnancy rate per stimulation significantly increased with the number of oocytes retrieved, the proportion of oocytes that developed into embryos of appropriate quality for transfer significantly decreased. A cycle efficiency index was introduced which expressed the rate of retrieved oocytes that developed into embryos of suitable quality for transfer or cryopreservation. The cycle efficiency index was inversely proportional to the number of oocytes retrieved [24].

Zhang and colleagues evaluated the oocyte utilization rate in Min stim IVF using a combination of 50 mg CC/d and 75U of hMG/d starting on cycle days 4 to 7 [25]. The metaphase II (MII) oocyte utilization rate was defined as the number of live births divided by the number of MII oocytes retrieved per pick up and utilized for transfer. In line with the previously shown study, an inverse relationship between oocyte utilization and oocytes retrieved was found. The utilization rate was 30.3% if 1–2 oocytes were retrieved, 9.3% if 3–6 oocytes, and 4.3% if ≥ 7 oocyte were retrieved (*p* < 0.05). However, due to the higher number of transferable embryos in cases with higher number of oocytes, cumulative clinical pregnancy and live birth rates were similar in all categories (*p* > 0.05) [25].

The retrospective analysis of Stoop and colleagues included 23.354 cIVF cycles, which were subdivided into three groups according to number of retrieved metaphase II oocytes: group 1, 1–5 oocytes; group 2, 6–10 oocytes; group 3, ≥ 11 oocytes [23]. One of the main outcomes of the study was the oocyte utilization rate, calculated as number of live births per metaphase II oocyte. Consistent with previous studies, the results showed that oocyte utilization decreased the number of oocytes retrieved. Specifically, in women aged 23–37 years, oocyte utilization rate was 1.7% higher in the 6–10 oocyte response group than in the 11 or more oocyte response group and 2.8% higher in the 1–5 MII oocyte response group, with age-adjusted absolute rate differences of 1.6% (95% CI 0.9–2.4%; *p* < 0.0001) and 2.8% (95% CI 2.1–3.5%; *p* < 0.001), respectively [23].

Inge also retrospectively assessed the cycle efficiency index [30]. Patients were subdivided into groups by oocyte yield (low, intermediate and high). Study results were similar to those described above. The pregnancy rate was comparable between groups, but patients with a low number of oocytes (1–5) required on average 9.6 oocytes per live birth, compared to 25.1 and 51.5 oocytes in those with an intermediate (6–16) and high (> 16) number of oocytes [30].

Even though the results of all four studies are in line with our study [23–25, 30], they present different concepts as they compared the oocyte development potential of the same stimulation protocols but did not compare different IVF therapies as in our study. Furthermore, as these four studies were performed more than 10 years ago, it cannot be excluded that more recent studies would have led to slightly different results due to improvements in laboratory techniques.

Still, all these studies provide cumulative evidence from different perspectives that increasing numbers of oocytes are associated with decreasing development potential of oocytes, expressed as lower fertilisation, embryo development and pregnancy rates [23–25, 30–32].

But what is the biological rationale behind these findings?

In natural cycles only 1–2 follicles mature. All other growing follicles degenerate due to the mid follicular phase decrease in FSH concentration induced by inhibin B secretion by the leading follicle that has reached a diameter of 12 mm [33]. When follicular growth is stimulated with high dose gonadotropins, FSH concentration does not decrease, and the natural follicular selection process does not occur. Therefore, it can be speculated that follicles with less competent oocytes might continue to grow and are also aspirated. This speculation is supported by studies which compared the hormone concentrations in follicular fluid derived from naturally matured compared to high dose stimulated follicles. These studies showed that follicular fluid testosterone, E2 and AMH concentrations are significantly reduced in follicles of high-dose stimulated cycles [34, 35]. This could indicate some degree of granulosa cell dysfunction, imposed by gonadotropin stimulation and would explain the lower pregnancy and live birth rates associated with lower follicular AMH concentrations [35]. Low follicular fluid AMH concentration is associated with lower life birth rates as shown by Ciepiela et al. [36]. The impact of stimulation on follicular endocrinology might also explain the reduced oocyte maturation rate, lower fertilization rate and lower embryo quality [4, 32] and the lower live birth rate in cIVF compared to NC-IVF cycles [2].

Can any clinical consequences be drawn from these findings?

Ours and other studies indicate that high stimulation doses seem to have a negative impact on the potential of oocytes to generate a pregnancy. In women with moderate ovarian or high ovarian reserve, this negative impact is clinically hardly relevant as the high number of retrieved oocytes and the embryo selection process overcompensates this negative effect. However, in women with low or very low ovarian response, it might have a clinically relevant effect due to the low number of retrieved oocytes. In such cases, favouring IVF treatment cycles with lower stimulation dosages might lead to equal or possibly even higher success rates per aspirated oocyte.

In conclusion, our study extends the body of literature on the effect of ovarian stimulation on the development potential of oocytes. The study shows that the development potential is lower in cIVF compared to NC-IVF and shows for the first time that the development potential of oocytes derived from Min Stim IVF lies between those from NC- and cIVF cycles.

However, although these findings are very interesting from a basic research perspective, their impact on clinical science is very difficult to assess, and we have to consider that early ovulation may also occur with NC-IVF. The overall impact of this finding on IVF success rates is difficult to estimate because pregnancy and live birth rates depend on a wide range of factors, and oocyte number is one of the most important, being positively associated with the stimulation dose.

## Supplementary Information

Below is the link to the electronic supplementary material.

Supplementary file1 (DOCX 41 KB)

Supplementary file2 (DOCX 22 KB)

Supplementary file3 (DOCX 19 KB)

Supplementary file4 Pairwise comparison of IVF protocols for the transition from oocyte to zygote, and for the transitions from zygote to gestational sac and live birth. Abbreviation: AI, aromatase inhibitor; CC, Clomiphene citrate; cIVF, conventional IVF; FSH, follicle stimulating hormone; hMG, human menopausal gonadotropin; IVF, in-vitro-fertilisation; NC-IVF, Natural cycle IVF. Estimated absolute risk difference along with the associated 95% confidence interval (in bracket) and p-value (in parentheses). A value lower than 0 represents an advantage of the protocol on the right. The cells are color-coded-based on the p-value, with more significant differences appearing in deeper shades of red. (PPTX 105 KB)

Supplementary file5 Pairwise comparison of protocols for the transition from zygote to gestational sac. Abbreviation: AI, aromatase inhibitor; CC, Clomiphene citrate; cIVF, conventional IVF; FSH, follicle stimulating hormone; hMG, human menopausal gonadotropin; IVF, in-vitro-fertilisation; NC-IVF, Natural cycle IVF. Estimated absolute risk difference along with the associated 95% confidence interval (in bracket) and p-value (in parentheses). A value lower than 0 represents an advantage of the protocol on the right. The cells are color-coded-based on the p-value, with more significant differences appearing in deeper shades of red. (PPTX 101 KB)

Supplementary file6 Pairwise comparison of protocols for the transition from zygote to live birth. Abbreviation: AI, aromatase inhibitor; CC, Clomiphene citrate; cIVF, conventional IVF; FSH, follicle stimulating hormone; hMG, human menopausal gonadotropin; IVF, in-vitro-fertilisation; NC-IVF, Natural cycle IVF. Estimated absolute risk difference along with the associated 95% confidence interval (in bracket) and p-value (in parentheses). A value lower than 0 represents an advantage of the protocol on the right. The cells are color-coded-based on the p-value, with more significant differences appearing in deeper shades of red. (PPTX 103 KB)

## Data Availability

The data underlying this article will be shared on reasonable request to the corresponding author.
