# Supplementary material for: Oocyte and zygote development potential in minimal stimulation, natural cycle and conventionally stimulated IVF: an international multi-centre retrospective cohort study
Source: J Assist Reprod Genet. 2025 May 28;42(7):2331–40. doi: 10.1007/s10815-025-03508-3 (PMC12356764; doi:10.1007/s10815-025-03508-3)
Supplement: Supplementary file 1 — Supplementary file1 (DOCX 41 KB) [file 10815_2025_3508_MOESM1_ESM.docx]

**Supplementary Table S1:** Cycles characteristics overall and stratified by IVF protocol

| **Characteristics** | **Overall** | **NC-IVF** | **CC-IVF** | **AI-IVF** | **hMG/**  **FSH-IVF** | **CC +**  **hMG/**  **FSH-IVF** | **AI +**  **hMG/**  **FSH-IVF** | **cIVF** |
| --- | --- | --- | --- | --- | --- | --- | --- | --- |
| **Number of cycles, n** | 4583 | 1483 | 434 | 149 | 153 | 200 | 272 | 1892 |
| **Number oocytes** |  |  |  |  |  |  |  |  |
| 0 | 470  (10%) | 285  (19%) | 53  (12%) | 24  (16%) | 24  (16%) | 17  (8.5%) | 20  (7.4%) | 47  (2.5%) |
| >0-≤1 | 1597 (35%) | 1110 (75%) | 186  (43%) | 67  (45%) | 43  (28%) | 47  (24%) | 43  (16%) | 101  (5.3%) |
| >1-≤2 | 565  (12%) | 83  (5.6%) | 150  (35%) | 49  (33%) | 44  (29%) | 40  (20%) | 61  (22%) | 138  (7.3%) |
| >2-≤48 | 1951  (43%) | 5  (0.3%) | 45  (10%) | 9  (6.0%) | 42  (27%) | 96  (48%) | 148  (54%) | 1606  (85%) |
| Median [IQR] | 2.0 [1.0,6.0] | 1.0 [1.0,1.0] | 1.0 [1.0,2.0] | 1.0 [1.0,2.0] | 2.0 [1.0,3.0] | 2.0 [1.0,4.0] | 3.0 [2.0,5.0] | 7.0 [4.0,12.0] |
| Mean (±SD) | 4.3 (±5.3) | 0.9 (±0.5) | 1.5 (±1.0) | 1.3 (±0.9) | 2.6 (±3.0) | 3.0 (±2.5) | 3.6 (±2.9) | 8.3 (±6.1) |
| **Fertilization method** |  |  |  |  |  |  |  |  |
| ICSI | 3125  (68%) | 926  (62%) | 286  (66%) | 78  (52%) | 117  (76%) | 123  (62%) | 147  (54%) | 1448  (77%) |
| IVF | 981  (21%) | 268  (18%) | 95  (22%) | 47  (32%) | 12  (7.8%) | 60  (30%) | 104  (38%) | 395  (21%) |
| None | 477  (10%) | 289  (19%) | 53  (12%) | 24  (16%) | 24  (16%) | 17  (8.5%) | 21  (7.7%) | 49  (2.6%) |
| **Number of zygotes** |  |  |  |  |  |  |  |  |
| 0 | 1117  (24%) | 593  (40%) | 143  (33%) | 67  (45%) | 41  (27%) | 39  (20%) | 54  (20%) | 180  (9.5%) |
| >0 - ≤1 | 1523 (33%) | 845  (57%) | 195  (45%) | 60  (40%) | 56  (37%) | 68  (34%) | 62  (23%) | 237  (13%) |
| >1 - ≤2 | 549  (12%) | 45  (3.0%) | 81  (19%) | 19  (13%) | 28  (18%) | 39  (20%) | 58  (21%) | 279  (15%) |
| >2 - ≤36 | 1394 (30%) | 0  (0%) | 15  (3.5%) | 3  (2.0%) | 28  (18%) | 54  (27%) | 98  (36%) | 1196  (63%) |
| Median [IQR] | 1.0  [1.0, 3.0] | 1.0 [0.0,1.0] | 1.0 [0.0,1.0] | 1.0 [0.0,1.0] | 1.0 [0.0,2.0] | 1.0 [1.0,3.0] | 2.0 [1.0,3.0] | 4.0 [2.0,6.0] |
| Mean (±SD) | 2.4 (±3.2) | 0.6 (±0.5) | 0.9 (±0.8) | 0.7 (±0.8) | 1.6 (±1.9) | 1.8 (±1.7) | 2.3 (±2.2) | 4.5 (±3.9) |
| **Number of cleavage embryos** |  |  |  |  |  |  |  |  |
| 0 | 1249  (27%) | 605  (41%) | 146  (34%) | 70  (47%) | 43  (28%) | 42  (21%) | 55  (20%) | 288  (15%) |
| >0 - ≤1 | 1529 (33%) | 834  (56%) | 199  (46%) | 57  (38%) | 55  (36%) | 66  (33%) | 63  (23%) | 255  (13%) |
| >1 - ≤2 | 553  (12%) | 44  (3.0%) | 77  (18%) | 19  (13%) | 30  (20%) | 38  (19%) | 58  (21%) | 287  (15%) |
| >2 - ≤12 | 1252 (27%) | 0  (0%) | 12  (2.8%) | 3  (2.0%) | 25  (16%) | 54  (27%) | 96  (35%) | 1062  (56%) |
| Median [IQR] | 1.0  [0.0, 3.0] | 1.0 [0.0,1.0] | 1.0 [0.0,1.0] | 1.0 [0.0,1.0] | 1.0 [0.0,2.0] | 1.0 [1.0,3.0] | 2.0 [1.0,3.0] | 3.0 [1.0,5.0] |
| Mean (±SD) | 1.9 (±2.2) | 0.6 (±0.5) | 0.9 (±0.8) | 0.7 (±0.8) | 1.5 (±1.7) | 1.8 (±1.7) | 2.1 (±1.8) | 3.3 (±2.7) |
| Occurrence of at least 1 embryo transfer | 3057  (67%) | 791  (53%) | 275  (63%) | 75  (50%) | 102  (67%) | 152  (76%) | 214  (79%) | 1448  (77%) |
| Unknown | 4 | 3 | 0 | 0 | 0 | 0 | 0 | 1 |
| **Number of transferred embryos** |  |  |  |  |  |  |  |  |
| 0 | 1522  (33%) | 689  (47%) | 159  (37%) | 74  (50%) | 51  (33%) | 48  (24%) | 58  (21%) | 443  (23%) |
| 1 | 2166 (47%) | 760  (51%) | 243  (56%) | 59  (40%) | 78  (51%) | 95  (48%) | 113  (42%) | 818  (43%) |
| 2 | 880  (19%) | 31  (2.1%) | 32  (7.4%) | 16  (11%) | 22  (14%) | 56  (28%) | 100  (37%) | 623  (33%) |
| 3 | 11  (0.2%) | 0  (0%) | 0  (0%) | 0  (0%) | 2  (1.3%) | 1  (0.5%) | 1  (0.4%) | 7  (0.4%) |
| Median [IQR] | 1.0  [0.0, 1.0] | 1.0 [0.0,1.0] | 1.0 [0.0,1.0] | 1.0 [0.0,1.0] | 1.0 [0.0,1.0] | 1.0 [1.0,2.0] | 1.0 [1.0,2.0] | 1.0 [1.0,2.0] |
| Mean (±SD) | 0.9 (±0.7) | 0.6 (±0.5) | 0.7 (±0.6) | 0.6 (±0.7) | 0.8 (±0.7) | 1.1 (±0.7) | 1.2 (±0.8) | 1.1 (±0.8) |
| Unknown | 4 | 3 | 0 | 0 | 0 | 0 | 0 | 1 |
| **Number of gestational sacs** |  |  |  |  |  |  |  |  |
| 0 | 3650 (80%) | 1277 (86%) | 377  (87%) | 130  (87%) | 129  (84%) | 155  (78%) | 204  (75%) | 1378  (73%) |
| 1 | 825  (18%) | 198  (13%) | 55  (13%) | 17  (11%) | 21  (14%) | 37  (19%) | 56  (21%) | 441  (23%) |
| 2 | 99  (2.2%) | 3  (0.2%) | 2  (0.5%) | 2  (1.3%) | 2  (1.3%) | 7  (3.5%) | 12  (4.4%) | 71  (3.8%) |
| 3 | 2  (<0.1%) | 0  (0%) | 0  (0%) | 0  (0%) | 1  (0.7%) | 0  (0%) | 0  (0%) | 1  (<0.1%) |
| Median [IQR] | 0.0  [0.0, 0.0] | 0.0 [0.0,0.0] | 0.0 [0.0,0.0] | 0.0 [0.0,0.0] | 0.0 [0.0,0.0] | 0.0 [0.0,0.0] | 0.0 [0.0,0.5] | 0.0 [0.0,1.0] |
| Mean (±SD) | 0.2 (±0.5) | 0.1 (±0.4) | 0.1 (±0.4) | 0.1 (±0.4) | 0.2 (±0.5) | 0.3 (±0.5) | 0.3 (±0.5) | 0.3 (±0.5) |
| Unknown | 7 | 5 | 0 | 0 | 0 | 1 | 0 | 1 |
| **Number of live births** |  |  |  |  |  |  |  |  |
| 0 | 3869 (85%) | 1329 (90%) | 387  (89%) | 133  (89%) | 134  (88%) | 172  (86%) | 222  (82%) | 1492  (79%) |
| 1 | 645  (14%) | 146 (9.9%) | 45  (10%) | 15  (10%) | 18  (12%) | 26  (13%) | 42(15%) | 353  (19%) |
| 2 | 61  (1.3%) | 3  (0.2%) | 2  (0.5%) | 1  (0.7%) | 1  (0.7%) | 1  (0.5%) | 8  (2.9%) | 45  (2.4%) |
| 3 | 1  (<0.1%) | 0  (0%) | 0  (0%) | 0  (0%) | 0  (0%) | 0  (0%) | 0  (0%) | 1  (<0.1%) |
| Median [IQR] | 0.0  [0.0, 0.0] | 0.0 [0.0,0.0] | 0.0 [0.0,0.0] | 0.0 [0.0,0.0] | 0.0 [0.0,0.0] | 0.0 [0.0,0.0] | 0.0 [0.0,0.0] | 0.0 [0.0,0.0] |
| Mean (±SD) | 0.2 (±0.4) | 0.1 (±0.3) | 0.1 (±0.3) | 0.1 (±0.3) | 0.1 (±0.4) | 0.1 (±0.4) | 0.2 (±0.5) | 0.2 (±0.5) |
| Unknown | 7 | 5 | 0 | 0 | 0 | 1 | 0 | 1 |
| **Number of miscarriages** |  |  |  |  |  |  |  |  |
| 0 | 4341 (95%) | 1428 (97%) | 424  (98%) | 145  (97%) | 147  (96%) | 177  (89%) | 251  (93%) | 1769  (94%) |
| 1 | 227 (5.0%%) | 50  (3.4%) | 10  (2.3%) | 4  (2.7%) | 5  (3.3%) | 22  (11%) | 20  (7.5%) | 116  (6.1%) |
| 2 | 7  (0.2%) | 0  (0%) | 0  (0%) | 0  (0%) | 0  (0%) | 0  (0%) | 1  (0.4%) | 6  (0.3%) |
| 3 | 1  (<0.1%) | 0  (0%) | 0  (0%) | 0  (0%) | 1  (0.7%) | 0  (0%) | 0  (0%) | 0  (0%) |
| Median [IQR] | 0.0  [0.0, 0.0] | 0.0 [0.0,0.0] | 0.0 [0.0,0.0] | 0.0 [0.0,0.0] | 0.0 [0.0,0.0] | 0.0 [0.0,0.0] | 0.0 [0.0,0.0] | 0.0 [0.0,0.0] |
| Mean (±SD) | 0.1 (±0.2) | 0.0 (±0.2) | 0.0 (±0.2) | 0.0 (±0.2) | 0.1 (±0.3) | 0.1 (±0.3) | 0.1 (±0.3) | 0.1 (±0.3) |
| Unknown | 7 | 5 | 0 | 0 | 1 | 0 | 0 | 1 |

Abbreviations: AI, aromatase inhibitor; CC, Clomiphene citrate; cIVF, conventional IVF; FSH, follicle stimulating hormone; hMG, human menopausal gonadotropin; ICSI, intracytoplasmic sperm injection; IQR, interquartile range; IVF, *in-vitro-fertilisation*; NC-IVF, Natural cycle IVF; SD, standard deviation.

Note: the unknown label for the “Number of transferred embryos”, “Unknown” refers to cycles in which cryopreserved embryos were transferred; the label “Unknown” for the “Number of gestational sacs”, “Number of live births” and “Number of miscarriages” refers to cycles in which cryopreserved embryos were transferred and cycles in which the number of gestational sacs, live births or miscarriage were unknown.
