# Supplementary material for: Oocyte and zygote development potential in minimal stimulation, natural cycle and conventionally stimulated IVF: an international multi-centre retrospective cohort study
Source: J Assist Reprod Genet. 2025 May 28;42(7):2331–40. doi: 10.1007/s10815-025-03508-3 (PMC12356764; doi:10.1007/s10815-025-03508-3)
Supplement: Supplementary file 2 — Supplementary file2 (DOCX 22 KB) [file 10815_2025_3508_MOESM2_ESM.docx]

**Table S2:** Logistic regression model for transitions probabilities

|  | **Oocyte 🡪 Zygote** | | | **Zygote 🡪 Gestational Sac** | | | **Zygote 🡪 Live birth** | | |
| --- | --- | --- | --- | --- | --- | --- | --- | --- | --- |
| **Characteristics** | **OR** | **95% CI** | **p-value** | **OR** | **95% CI** | **p-value** | **OR** | **95% CI** | **p-value** |
| **IVF protocols** |  |  | **<0.001** |  |  | **<0.001** |  |  | **<0.001** |
| NC-IVF | - | - |  | - | - |  | - | - |  |
| CC-IVF | 0.69 | 0.55, 0.87 |  | 0.59 | 0.41,  0.84 |  | 0.67 | 0.45,  1.00 |  |
| AI-IVF | 0.46 | 0.33, 0.66 |  | 0.87 | 0.49, 1.53 |  | 1.00 | 0.53, 1.86 |  |
| hMG/FSH- IVF | 0.69 | 0.52, 0.93 |  | 0.57 | 0.36, 0.93 |  | 0.55 | 0.32, 0.97 |  |
| CC + hMG/  FSH- IVF | 0.64 | 0.50, 0.82 |  | 0.70 | 0.48, 1.03 |  | 0.49 | 0.30, 0.79 |  |
| AI + hMG/  FSH- IVF | 0.66 | 0.53, 0.82 |  | 0.63 | 0.45, 0.88 |  | 0.59 | 0.40, 0.87 |  |
| cIVF | 0.44 | 0.38, 0.51 |  | 0.36 | 0.29,  0.44 |  | 0.35 | 0.28,  0.45 |  |
| **AMH level (ng/ml)** |  |  | **0.7** |  |  | **0.025** |  |  | **0.002** |
| <1 | - | - |  | - | - |  | - | - |  |
| ≥1 - <2 | 1.01 | 0.88, 1.15 |  | 1.09 | 0.86, 1.38 |  | 1.15 | 0.88,  1.51 |  |
| ≥2 | 0.97 | 0.86, 1.09 |  | 0.84 | 0.68, 1.04 |  | 0.78 | 0.61,  1.00 |  |
| **Fertilization method** |  |  | **0.054** |  |  | **0.6** |  |  | **0.4** |
| ICSI | - | - |  | - | - |  | - | - |  |
| IVF | 0.89 | 0.79, 1.00 |  | 0.94 | 0.75, 1.17 |  | 0.89 | 0.69, 1.15 |  |
| **Duration of infertility (years)** | 0.99 | 0.97, 1.01 | **0.2** | 0.98 | 0.94, 1.01 | **0.2** | 0.96 | 0.92, 1.00 | **0.071** |
| **Cause of infertility** |  |  | **0.003** |  |  | **>0.9** |  |  | **0.5** |
| Female & male | - | - |  | - | - |  | - | - |  |
| Female factor | 1.20 | 1.04, 1.39 |  | 0.98 | 0.75, 1.27 |  | 0.98 | 0.73, 1.33 |  |
| Male factor | 0.94 | 0.84, 1.05 |  | 0.98 | 0.79, 1.22 |  | 1.00 | 0.78, 1.28 |  |
| Idiopathic | 1.03 | 0.89, 1.20 |  | 0.92 | 0.70, 1.22 |  | 0.79 | 0.57, 1.11 |  |
| **BMI** | 0.99 | 0.98, 1.00 | **0.053** | 1.01 | 1.00, 1.03 | **0.2** | 1.01 | 0.99,  1.03 | **0.2** |
| **Women`s age (year)** | 0.99 | 0.98, 1.00 | **0.3** | 0.94 | 0.93,  0.96 | **<0.001** | 0.92 | 0.89,  0.94 | **<0.001** |

Abbreviations: AI, aromatase inhibitor; AMH, Anti Mullerian hormone; BMI, Body-Mass-Index; CI, Confidence interval; CC, Clomiphene citrate; cIVF, conventional IVF; FSH, follicle stimulating hormone; hMG, human menopausal gonadotropin; ICSI, intracytoplasmic sperm injection; IQR, interquartile range; IVF, *in-vitro-fertilisation*; NC-IVF, Natural cycle IVF; OR, Odd Ratio; SD, standard deviation.
