# Supplementary material for: Oocyte and zygote development potential in minimal stimulation, natural cycle and conventionally stimulated IVF: an international multi-centre retrospective cohort study
Source: J Assist Reprod Genet. 2025 May 28;42(7):2331–40. doi: 10.1007/s10815-025-03508-3 (PMC12356764; doi:10.1007/s10815-025-03508-3)
Supplement: Supplementary file 3 — Supplementary file3 (DOCX 19 KB) [file 10815_2025_3508_MOESM3_ESM.docx]

**Table S3:** cIVF: Logistic regression model for the transition probabilities in cycles with and without cryopreservation of zygote(s) and embryo(s)

|  | **Zygote 🡪 Gestational Sac** | | | **Zygote 🡪 Live birth** | | |
| --- | --- | --- | --- | --- | --- | --- |
| **Characteristics** | **OR** | **95% CI** | **p-value** | **OR** | **95% CI** | **p-value** |
| **AMH level (ng/ml)** |  |  | **0.009** |  |  | **0.003** |
| <1 | - | - |  | - | - |  |
| ≥1 - <2 | 0.89 | 0.65, 1.22 |  | 0.98 | 0.68, 1.42 |  |
| ≥2 | 0.68 | 0.51, 0.90 |  | 0.64 | 0.46, 0.90 |  |
| **Cryopreservation of zygote(s)** |  |  | **0.2** |  |  | **0.6** |
| No | - | - |  | - | - |  |
| Yes | 0.85 | 0.66, 1.10 |  | 0.92 | 0.68, 1.24 |  |
| **Cryopreservation of embryo(s)** |  |  | **<0.001** |  |  | **0.002** |
| No | - | - |  | - | - |  |
| Yes | 1.61 | 1.29, 2.01 |  | 1.51 | 1.17, 1.96 |  |
| **Fertilization method** |  |  | **0.7** |  |  | **0.8** |
| ICSI | - | - |  | - | - |  |
| IVF | 1.07 | 0.79, 1.43 |  | 0.96 | 0.67, 1.38 |  |
| **Duration of infertility (years)** | 0.99 | 0.95, 1.04 | **0.8** | 0.97 | 0.92, 1.03 | **0.4** |
| **Cause of infertility** |  |  | **0.2** |  |  | **0.2** |
| Female & male | - | - |  | - | - |  |
| Female factor | 1.26 | 0.87, 1.80 |  | 1.25 | 0.81, 1.93 |  |
| Male factor | 1.39 | 1.04, 1.85 |  | 1.43 | 1.01, 2.01 |  |
| Idiopathic | 1.17 | 0.79, 1.75 |  | 1.08 | 0.66, 1.74 |  |
| **BMI** | 1.02 | 1.00, 1.04 | **0.10** | 1.02 | 0.99, 1.04 | **0.2** |
| **Women`s age (years)** | 0.95 | 0.92, 0.97 | **<0.001** | 0.93 | 0.91, 0.96 | **<0.001** |

Abbreviations: AMH, Anti Mullerian hormone; BMI, Body-Mass-Index; CI, Confidence interval; cIVF, conventional IVF; ICSI, intracytoplasmic sperm injection; IVF, *in-vitro-fertilisation*; OR, Odds Ratio. Note: NO= no cryopreservation; YES = cryopreservation.
