# Supplementary figures and images for: Oocyte and zygote development potential in minimal stimulation, natural cycle and conventionally stimulated IVF: an international multi-centre retrospective cohort study
Source: J Assist Reprod Genet. 2025 May 28;42(7):2331–40. doi: 10.1007/s10815-025-03508-3 (PMC12356764; doi:10.1007/s10815-025-03508-3)

## Slide 1
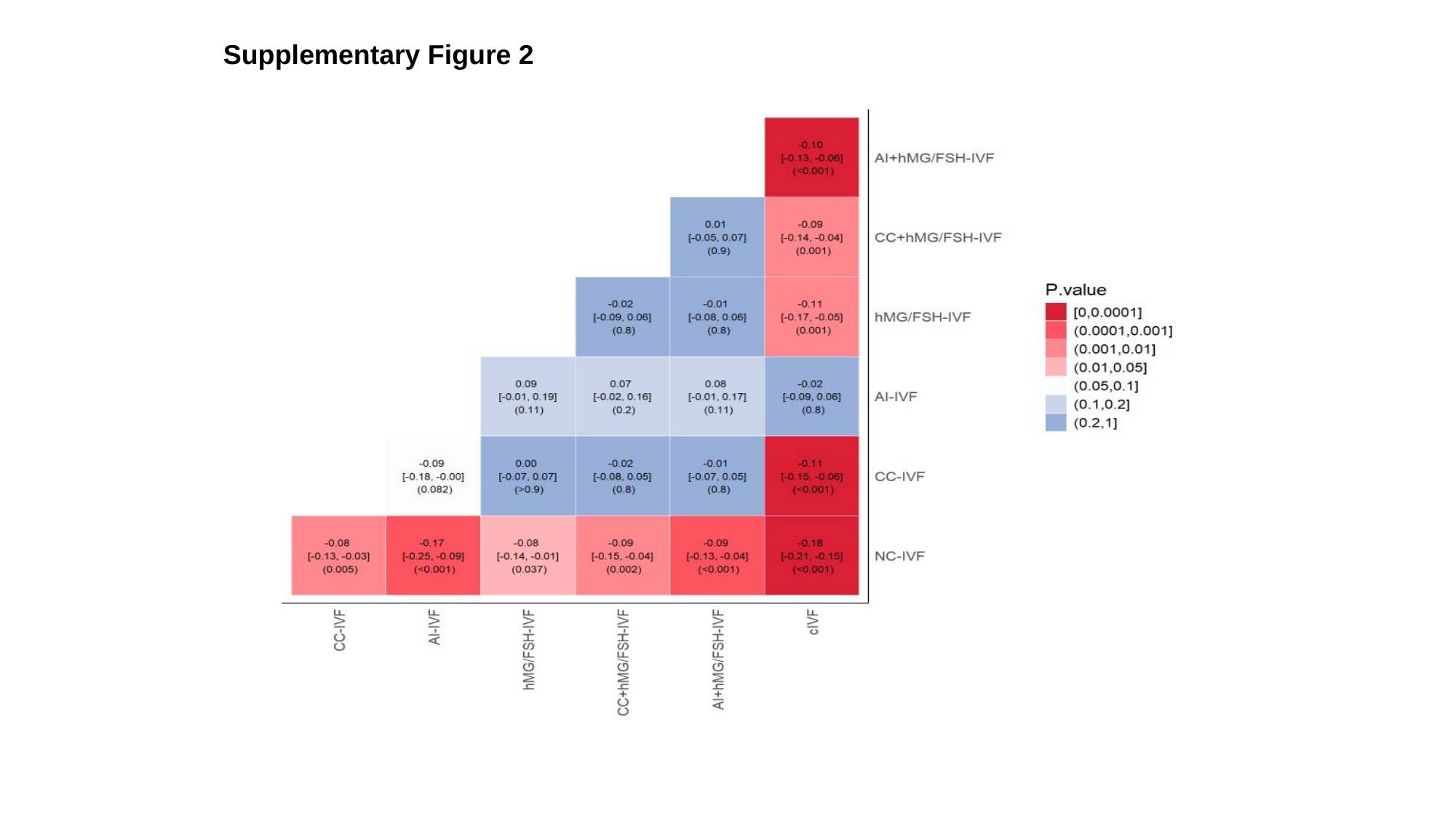

Supplementary Figure 2

Supplement: Supplementary file 4 — Supplementary file4 Pairwise comparison of IVF protocols for the transition from oocyte to zygote, and for the transitions from zygote to gestational sac and live birth. Abbreviation: AI, aromatase inhibitor; CC, Clomiphene citrate; cIVF, conventional IVF; FSH, follicle stimulating hormone; hMG, human menopausal gonadotropin; IVF, in-vitro-fertilisation; NC-IVF, Natural cycle IVF. Estimated absolute risk difference along with the associated 95% confidence interval (in bracket) and p-value (in parentheses). A value lower than 0 represents an advantage of the protocol on the right. The cells are color-coded-based on the p-value, with more significant differences appearing in deeper shades of red. (PPTX 105 KB) [file 10815_2025_3508_MOESM4_ESM.pptx]

## Slide 1
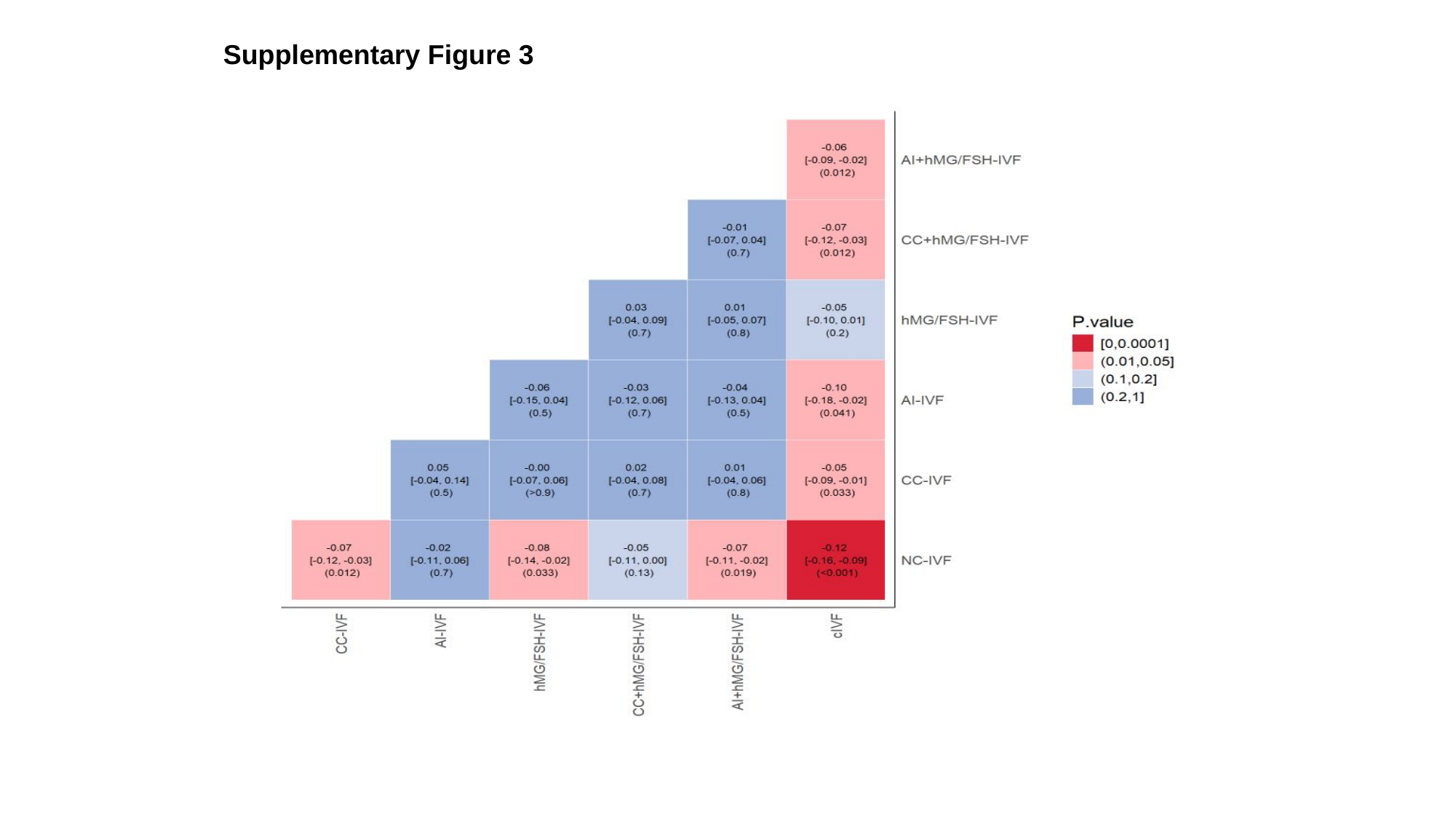

Supplementary Figure 3

Supplement: Supplementary file 5 — Supplementary file5 Pairwise comparison of protocols for the transition from zygote to gestational sac. Abbreviation: AI, aromatase inhibitor; CC, Clomiphene citrate; cIVF, conventional IVF; FSH, follicle stimulating hormone; hMG, human menopausal gonadotropin; IVF, in-vitro-fertilisation; NC-IVF, Natural cycle IVF. Estimated absolute risk difference along with the associated 95% confidence interval (in bracket) and p-value (in parentheses). A value lower than 0 represents an advantage of the protocol on the right. The cells are color-coded-based on the p-value, with more significant differences appearing in deeper shades of red. (PPTX 101 KB) [file 10815_2025_3508_MOESM5_ESM.pptx]

## Slide 1
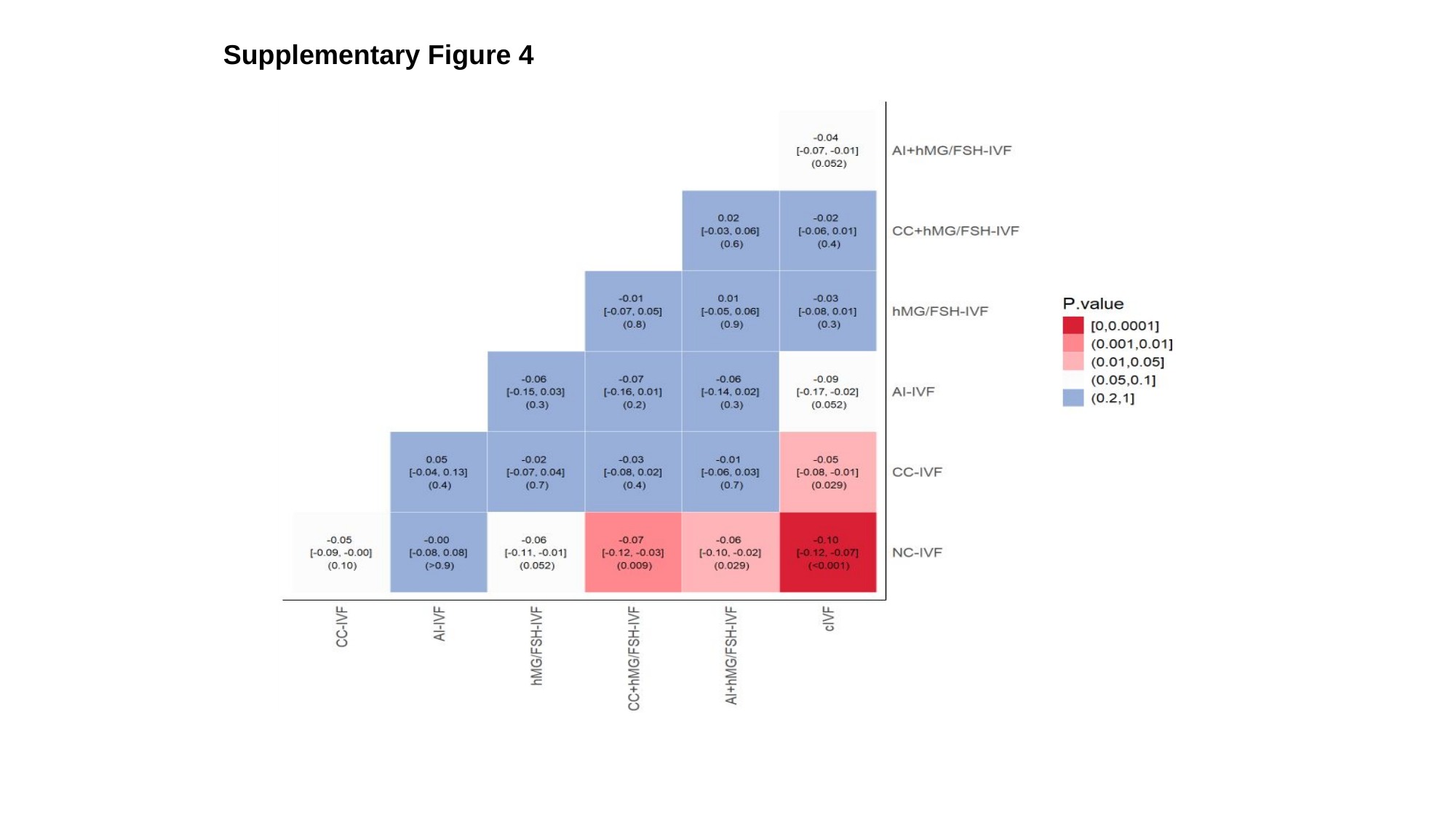

Supplementary Figure 4

Supplement: Supplementary file 6 — Supplementary file6 Pairwise comparison of protocols for the transition from zygote to live birth. Abbreviation: AI, aromatase inhibitor; CC, Clomiphene citrate; cIVF, conventional IVF; FSH, follicle stimulating hormone; hMG, human menopausal gonadotropin; IVF, in-vitro-fertilisation; NC-IVF, Natural cycle IVF. Estimated absolute risk difference along with the associated 95% confidence interval (in bracket) and p-value (in parentheses). A value lower than 0 represents an advantage of the protocol on the right. The cells are color-coded-based on the p-value, with more significant differences appearing in deeper shades of red. (PPTX 103 KB) [file 10815_2025_3508_MOESM6_ESM.pptx]
